# Gruppo Otologico’s Experience in Managing the So-Called Inoperable Tympanojugular Paraganglioma

**DOI:** 10.3390/brainsci14080745

**Published:** 2024-07-25

**Authors:** Mario Sanna, Mohammed Al-Khateeb, Melcol Hailu Yilala, Mohanad Almashhadani, Giuseppe Fancello

**Affiliations:** 1 Department of Otology and Skull Base Surgery, Gruppo Otologico, 29121 Piacenza, PC, Italygiuseppe.fancello91@gmail.com (G.F.); 2 Department of ORL-HNS, Rizgary Teaching Hospital, Erbil 44001, Iraq; 3 Department of ORL-HNS, School of Medicine, Addis Ababa University, Addis Ababa 9086, Ethiopia; melcol.hailu@aau.edu.et; 4 Department of ORL-HNS, Al-Yarmouk Teaching Hospital, Baghdad 10011, Iraq; mohanadhns80@gmail.com

**Keywords:** tympanojugular paraganglioma, complex tympanojugular PGL, ITFA, carotid artery stenting, preoperative balloon occlusion (PBO), vertebral artery closure

## Abstract

**Objective**: to identify advanced or “so-called inoperable” cases of tympanojugular paragangliomas (PGLs) and analyze how each case is surgically managed and followed afterward. **Study Design**: a retrospective case series study. **Methods:** Out of 262 type C and D TJPs and more than 10 cases of advanced or so-called inoperable cases, files of 6 patients with a diagnosis of advanced tympanojugular PGLs who were referred to an otology and skull-base center between 1996 and 2021 were reviewed to analyze management and surgical outcomes. The criteria for choosing these cases involve having one or more of the following features: (1) a large-sized tumor; (2) a single ipsilateral internal carotid artery (ICA); (3) involvement of the vertebral artery; (4) a considerable involvement of the ICA; (5) an extension to the clivus, foramen magnum, and cavernous sinus; (6) large intradural involvement (IDE); and (7) bilateral or multiple PGLs. **Results**: The age range at presentation was 25–43 years old, with a mean of 40.5 years: two females and four males. The presenting symptoms were glossal atrophy, hearing loss, pulsatile tinnitus, dysphonia, shoulder weakness, and diplopia. The modified Infratemporal Fossa Approach (ITFA) with a transcondylar–transtubercular extension is the principal approach in most cases, with additional approaches being used accordingly. **Conclusions:** The contemporary introduction of carotid artery stenting with the direct and indirect embolization of PGLs has made it possible to operate on many cases, which was otherwise considered impossible to treat surgically. Generally, the key is to stage the removal of the tumor in multiple stages during the management of complex PGLs to decrease surgical morbidities. A crucial aspect is to centralize the treatment of PGLs in referral centers with experienced surgeons who are trained to plan the stages and manage possible surgical complications.

## 1. Introduction

Tympanojugular paragangliomas (TJPs) are rare locally aggressive neoplasms of the temporal bone and skull base that arise from the adventitia of the jugular bulb [1]. Because TPJ tumors grow slowly and silently, they often present at later stages, making cases with extensive involvement inoperable in the past.

A TJP presents a challenge to the treating physician because of its high vascularity, locally aggressive nature, and extension and involvement of important neurovascular structures such as the jugular bulb (JB), facial nerve (FN), internal carotid artery (ICA), and lower cranial nerves (LCNs) [1].

Advances in neuroradiology, endovascular interventions, lateral skull-base approaches, neuroanesthesia, and postoperative care make surgical removal a safe and preferred way of treatment [2,3,4,5]. Thus, gross total removal remains the mainstay of treatment even in advanced and complex lesions [6].

Some cases, however, are still complex and challenging to treat and require treatment in the hands of highly skilled surgeons in specialized centers. Examples of such cases include large tumors (modified Fisch class C3–4, D, and V); tumors with large intradural extension; tumors involving the cavernous sinus, clivus, and foramen magnum; tumors involving the ICA and vertebral arteries; previously irradiated tumors; recurrent tumors; tumors involving a single carotid artery; bilateral and multiple tumors; and tumors on the side of the dominant or single sigmoid sinus [2,7,8,9,10,11,12].

A thorough preoperative evaluation and individualized surgical planning are mandatory prerequisites in managing complex and so-called “inoperable” TJPs. A preoperative evaluation of such cases includes a complete otoneurologic clinical examination with an emphasis on the assessment of the function of FN and LCNs, pure-tone audiometry, high-resolution temporal bone CT scans (HRCTs), gadolinium-enhanced magnetic resonance imaging (MRI), and angiography with both arterial and venous phases and four-vessel angiography with cross-compression tests [1,2,5].

In all the cases of complex or so-called “inoperable” TJPs, preoperative embolization is performed 48 h before surgery. Endovascular intervention, such as stenting or permanent balloon occlusion (PBO), however; can be used depending on the presence of indication during the initial MRI and angiography evaluation. Examples of such indications include an encasement of the distal cervical, vertical, and horizontal segments of the ICA between 270 and 360 degrees; evidence of stenosis and irregularity of the arterial lumen; extensive blood supply from branches of ICA; prior irradiation; and past surgery including ICA manipulation [13].

Conversely, extensive involvement of ICA doesn’t represent an absolute contraindication to surgery. Therefore, when the tumor invades the ICA and there is adequate collateral blood flow, PBO is carried out. However, we frequently perform intraluminal stenting two to three months prior to surgery in instances with inadequate collateral blood flow [1,13].

Achieving proximal and distal control of the major vessels and maximizing structural exposure while avoiding damage are the objectives of surgical therapy in these instances. Therefore, a surgical strategy that maximizes exposure while minimizing morbidity should be pursued. Since its description by Fisch and Pillsbury in 1979 and its modification by Sanna, the Infratemporal Fossa Approach (ITFA) has come to be recognized as the preferred method for the surgical therapy of TJPs [14,15].

The surgical management and results of several TJP cases that were previously deemed complicated and “inoperable” are presented in this article.

## 2. Material and Methods

The medical records of patients who have been diagnosed with a TJP at Gruppo Otologico Piacenza-Rome, between 1996 and 2021 were thoroughly examined.

Table 1 shows the updated Fisch classification of TJ PGLs that was used to classify all tumors [16]. The gathered information was examined for age, gender, symptoms at presentation, surgical techniques, tumor size and location as established by radiologic and surgical findings, surgical results, and clinical and radiologic follow-up observations.

A thorough otologic and neurologic examination was performed on each subject. The House–Brackmann (HB) grading system of FN was used to grade the facial function prior to the procedure, right after the procedure, and at each follow-up appointment [17]. In our series, gadolinium-enhanced MRIs and HRCT with bone windows were performed on all patients. In order to investigate the arterial supply, venous circulation, and blood flow of the sigmoid sinus and jugular bulb, arteriovenous magnetic resonance imaging and four-vessel angiography were carried out. A preoperative embolization of the tumor was performed 24 to 48 h before surgery using polyvinyl alcohol. An ICA stent had to be inserted in three patients. Pure-tone audiometry was used to measure hearing levels in each case, and flexible fiber optic naso-pharyngo-laryngoscopy was performed.

## 3. Intraoperative Preparation

The surgical site is shaved following the patient’s placement. The patient is placed in a supine position with the head turned to the other side for each approach (Supplementary Materials). Deep vein thrombosis is avoided by using stockings. To enable continuous electromyographic monitoring of the facial nerve, pairs of electrodes are inserted in the orbicularis oculi and orbicularis oris muscles. The surgical area is then prepared with 10 ppm of ether and Citrosil. Furthermore, the abdominal area is cleaned and draped in a similar manner in order to take a piece of fat which is used to obliterate the surgical cavity at the end of the procedure.

## 4. Postoperative Care

Once the surgery is complete, a tight bandage is applied and left in place for four to five days. There are no drains implanted during intradural operations. 

After that, the patients are transferred to the critical care unit, where they will be closely followed for changes in blood pressure, pulse rate, respiratory rate, arterial oxygen saturation, and ECG tracing. Furthermore, the patient’s degree of consciousness, pupillary reflexes, and motor responses are assessed every 15 min for the first six hours; then after every half hour for the next 12 to 18 h.

The patients are moved to the ward after 24 h where the vital signs and state of consciousness continue to be monitored. Subsequently, the indwelling urinary catheter is removed and if there is adequate swallowing function the nasogastric tube can be withdrawn, and oral fluids can be initiated. Finally, in order to reduce the risk of pulmonary embolism, early ambulation is advised following the first 24 h.

## 5. Clinical Cases with Illustrations

### 5.1. Case 1: (C4Di2)

**Figure 1:**
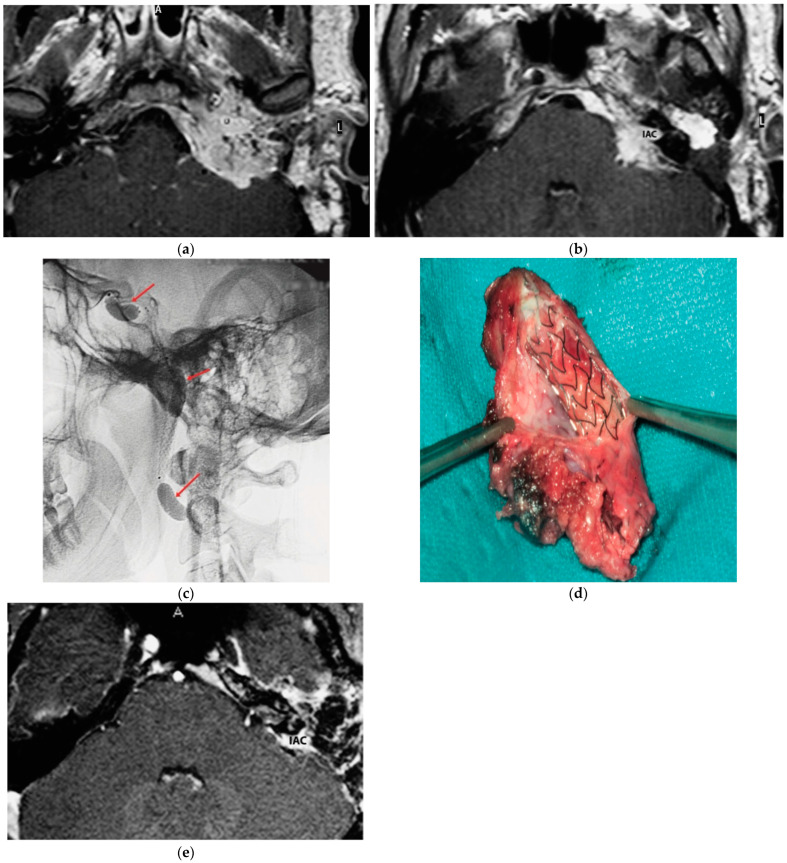
(Case 1) (**a**) Axial-enhanced T1W MRI, with large intradural tumor extension. (**b**) Axial-enhanced T1W MRI, with extensive involvement of posterior fossa dura with intradural and IAC involvement. (**c**) Plain lateral view skull X-ray. Red arrows indicate balloons during permanent occlusion of the IAC. (**d**) Intra-carotid stent was seen after opening the wall of the carotid artery. (**e**) Axial-enhanced T1W MRI revealing dural infiltration and the involvement of IAC.

A 25-year-old male patient with a persistent left TJP was sent to our center following three previous operations performed elsewhere. On admission, he presented with profound hearing loss with LCN palsy. The latest radiological examinations showed a C4Di2 TJ PGL. As there was significant involvement of the ICA, an intra-ICA stent was placed. However, a preoperative angiography showed remarkable residual vascular supply from the ICA, which could not be embolized. One contributing factor was that the stent diameter was inadequate to occlude the vascular supply from the affected ICA. Thus, the decision was made to proceed with PBO after confirming adequate contralateral supply. A first-stage surgery was performed through revision ITFA-A with extradural tumor removal including involved ICA. By using a combination of a translabyrinthine (TLAB) and petro-occipital-trans-sigmoid (POTS) approach, the remnant intradural tumor was removed in the second stage, along with the internal auditory canal (IAC) bone and its contents that the tumor had infiltrated (Figure 1). Three years after the second-stage operation, sural nerve grafting was performed for reconstruction of the FN function with HB-IV in the last follow-up.

### 5.2. Case 2: (C3Di1 + Stage I VP)

A 40-year-old male patient [18] was admitted to our center with the diagnosis of right vagal PGL and TJP. The fact that paragangliomas run in his family has been confirmed with genetic tests. The patient underwent surgery at a different facility fifteen years before a left carotid body paraganglioma (CBP), which led to ligation of the left common carotid artery. A clinical examination demonstrated the involvement of right LCN with vocal cord paralysis that was well-compensated.

**Figure 2:**
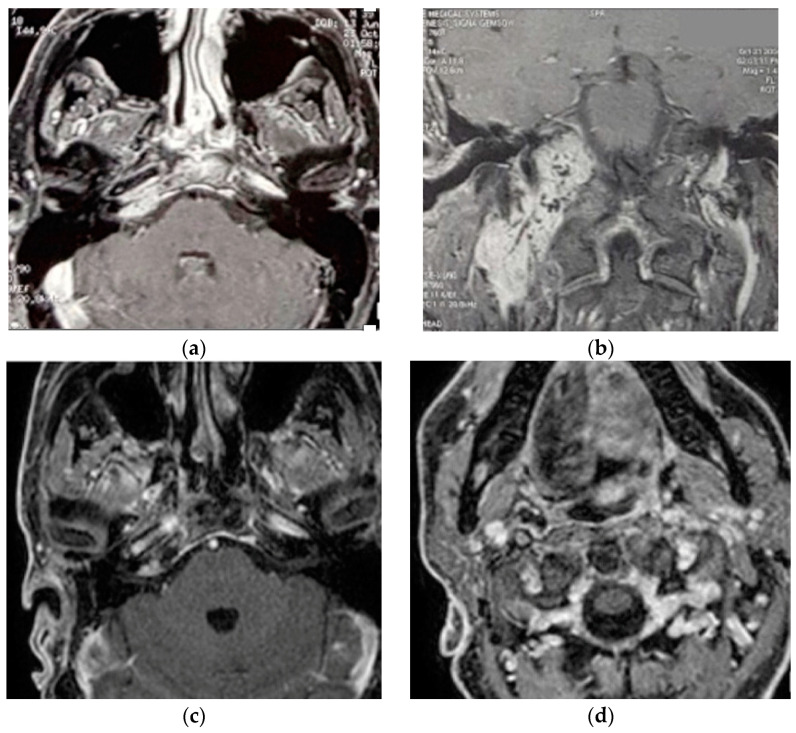
(Case 2) (**a**,**b**) Enhanced-axial T1W MRI showing C3Di1 + stage I VP. (**c**,**d**) Postoperative axial and coronal MRI showing no residual tumor.

Two separate masses at the level of the parapharyngeal space and jugular foramen, as well as the involvement of the vertical and horizontal parts of the petrous segment of the ICA, were shown by HRCT and gadolinium-enhanced MRIs (Figure 2). These findings are suggestive of a TJP (class C3Di1) and a VP (stage I). On the other hand, an angiography revealed a left ICA and common carotid artery blockage. The tumor encompassed the distal cervical section and the vertical part of the petrous segment of the right ICA. Therefore, seven weeks before the procedure, the patient had the only right ICA stented. He underwent surgery with an IFTA-A two days after embolization, and total excision was completed successfully and with the preservation of the ICA. After two years of surgery, the right FN function improved from HB-VI in the early postoperative period to HB-III. Contralateral compensation allowed for a good level of tolerance for the paralysis of the right vocal cord. For the first five years, the patient had annual HRCT and MRI scans; after that, they were performed roughly every two years. In October 2021, the last MRI and HRCT with contrast revealed no evidence of tumor recurrence.

### 5.3. Case 3: (C4Di2Vi)

**Figure 3:**
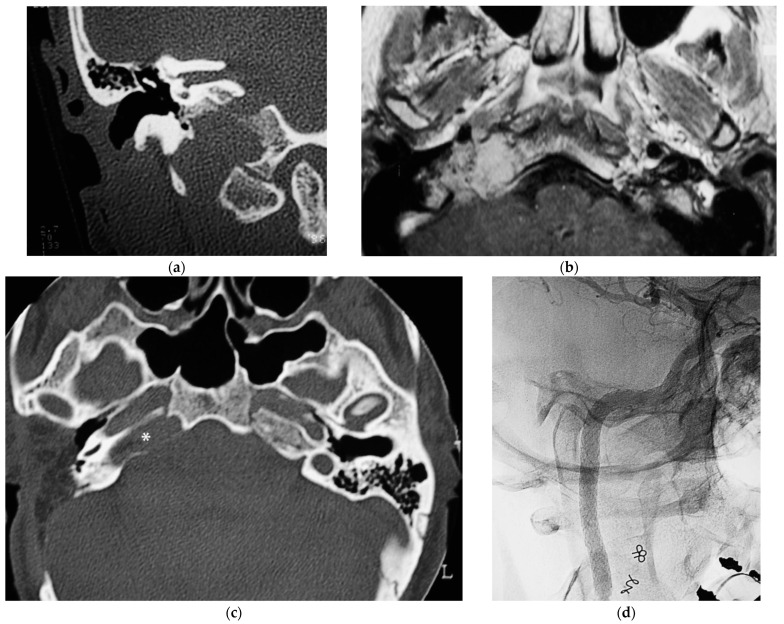

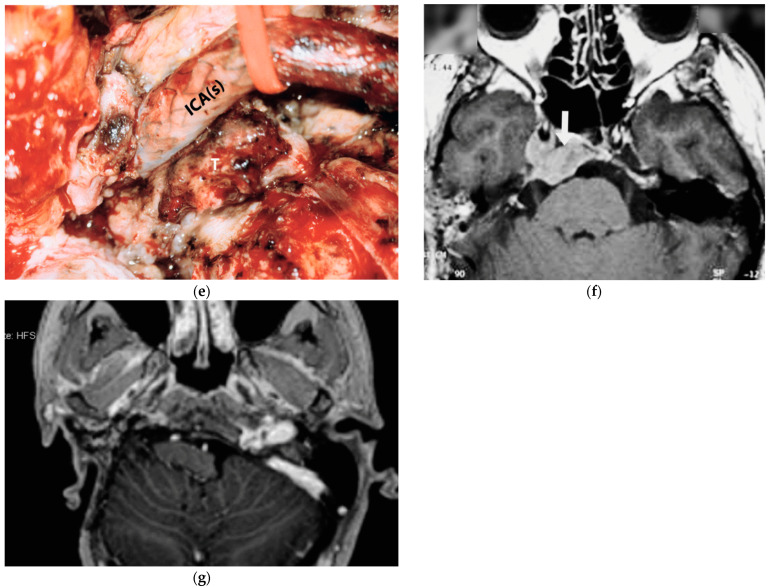
(Case 3) (**a**) CT scan, coronal view, of tumor extending into the craniocervical junction. (**b**) Axial-enhanced T1W MRI showing the tumor extension into the vertical segment of ICA. (**c**) Axial view, CT, after 1st stage showing residual tumor around horizontal ICA and PA. (**d**) X-ray screen shows ICA stenting at the level of foramen lacerum. (**e**) Stented ICA after removal of tumor-invaded adventitia. (**f**) Axial-enhanced T1W MRI showing residual tumor in the cavernous sinus. (**g**) Enhanced T1W MRI after 4th stage [1].

A 32-year-old woman affected by a TJP (class C4Di2Vi) on the right side was brought to the authors’ attention (Figure 3). Due to the tumor’s invasion of Dorello’s canal, the patient suffered from paralysis of the sixth cranial nerve. Because there was no contralateral compensation, the PBO test was unsuccessful.

First stage: To minimize the danger of intraoperative arterial rupture, a staged subtotal resection was planned, leaving some tumor remnants at the level of the ICA and at the level of the cavernous sinus to preserve ocular mobility. The first stage was carried out in October 1996 using an IFTA-A following preoperative embolization. During the postoperative phase, the FN function declined to HB-IV. Adjuvant treatment with stereotactic radiation was used for cavernous sinus involvement.

During the second stage, in March 1998, the intradural component of the tumor was excised using a translabyrinthine technique with transapical extension. It seems that the tumor invaded every dura that covers the posterior surface of the petrous bone. A follow-up CT scan revealed that the residual lesion was still present at the ICA level, but there was no tumor growth in the cavernous sinus. All the same, a residual lesion around the ICA grew more noticeable during follow-up surveillance. Since closing the artery was not possible, this was handled conservatively.

Third stage: After the ICA was stented and a fresh embolization was carried out in January 2004, new prospects emerged with the potential to use a stent to fortify the ICA’s vascular walls [13]. Subadventitial dissection along the vertical and horizontal segments was necessary for the treatment of the ICA.

Fourth stage: After 8 months from the last operation, the intradural vertebral artery was affected, as determined by an angiography and MRI. As a result, the tumor was completely excised with the involved VA by an extreme lateral approach. HRCT and MRI follow-ups for over 20 years showed no recurrence.

### 5.4. Case 4: (C3Di2 + Stage II Vagal PGL)

A 53-year-old female patient arrived at our center with the diagnosis of a left-side vagal and TJ PGL. Upon examination of the cranial nerves (CNs), left X and XII CN palsy was found. She has a dead ear on her left side, as determined by the pure-tone audiogram. The radiological examination demonstrated a Fisch stage II vagal PGL and a left-sided modified Fisch class C3Di2 TJ PGL [15]. Severe stenosis and anterior displacement of the cervical ICA were observed by angiography. Well-compensated collateral circulation was found by the preoperative occlusion test. Since there was a genuine risk of an ICA rupture during stent insertion due to severe stenosis, PBO was chosen over stent implantation.

The procedure was delayed for one month in order to give the cerebral vasculature time to adjust to the new hemodynamic conditions. IFTA-A was carried out during the first stage after super-selective embolization and planned subtotal tumor excision. For the second-stage surgery, the bony labyrinth was preserved as a landmark. LCNs with tumor infiltration were sacrificed. A periosteal invasion of the tumor necessitated extensive bone resection from the horizontal segment to the foramen lacerum. The ICA was removed right above the carotid bifurcation to the foramen lacerum. During the first stage, vagal paraganglioma and the extradural portion of the TJP were excised concurrently. After the first-stage surgery, an MRI revealed a remaining intradural component of the tumor. The intradural component of the tumor was entirely excised during the second step of the surgery and was performed using the POTS technique [15]. Following the second round of surgery, the postoperative radiological test revealed the absence of any remaining tumor. The swallowing function was successfully compensated for, and the FN function returned to HB grade III. After four years, there was still no indication of a return.

### 5.5. Case 5: (C3Di2Vi)

**Figure 4:**
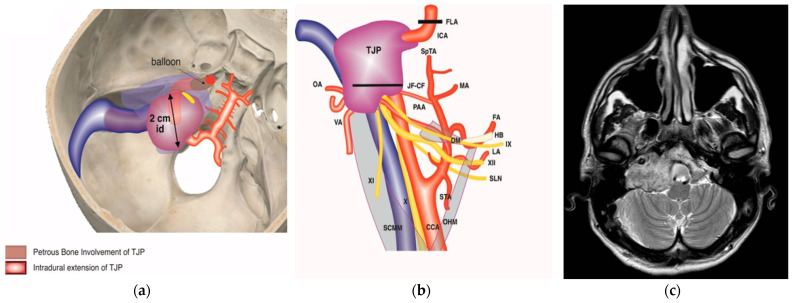

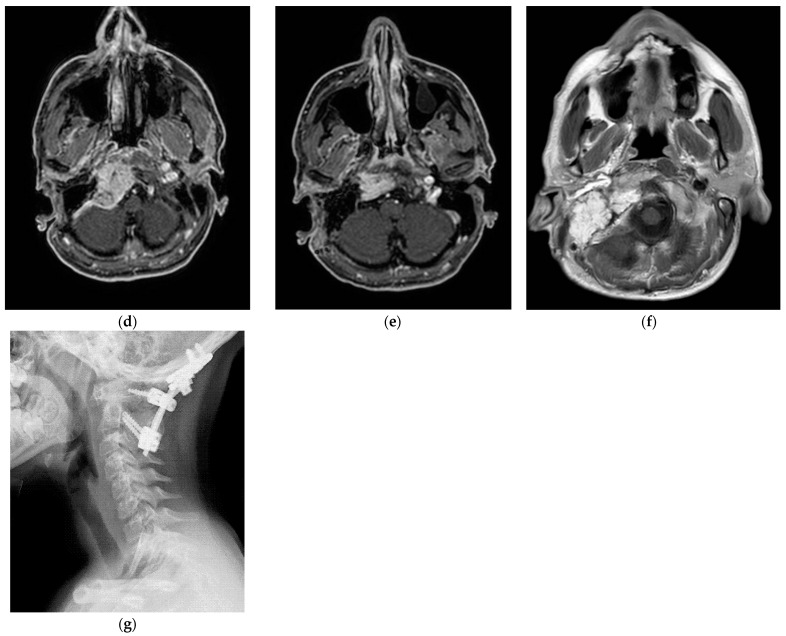
(Case 5) (**a**,**b**) Right tympanojugular paraganglioma (C3Di2Vi) involving the clivus, the vertebral artery, the foramen magnum, and the occipital condyle. (**c**) Axial-enhanced T1-weighted magnetic resonance imaging (MRI) shows a large mass extending to the intradural space up to the foramen magnum. (**d**) Axial-enhanced T1 MRI after first-stage tumor removal. (**e**) Enhanced T1 MRI after second-stage surgery. (**d**,**f**) Axial-enhanced T1 MRI after third-stage tumor removal, which shows total tumor removal with obliteration of the surgical cavity with abdominal fat. (**g**) A sagittal CT scan after total tumor removal during the third stage shows cervical–occipital fixation.

The fifth case was diagnosed with a right TJP (C3Di2Vi) at another center that involved the clivus, the vertebral artery, the foramen magnum, and the occipital condyle and was treated with embolization and radiotherapy. On admission, an MRI showed an enlarging tumor and a clinical examination revealed that there was paralysis of the cranial nerves X, XI, and XII (Figure 4).

The management plan was to stage the tumor removal to reduce the risk of surgical complications, and the occlusion test revealed good compensation and a preoperative PBO of the ICA and vertebral artery. At the first surgical stage in 2021, the tumor was removed at the jugular foramen, including the ICA, from the carotid bifurcation to the horizontal intra-petrous part through ITFA-A, leaving the tumor at the occipital condyle, clivus, and foramen magnum as well as the intradural part for the second stage. During the second stage, which was carried out in September 2022, the residual tumor from the occipital condyle, clivus, and foramen magnum was removed through revision ITFA-A with transcondylar extension. In February 2023, the third-stage procedure involved the removal of the clival and dural tumors using a trans-cochlear method. Additionally, the clivus was drilled until healthy bone appeared, and concurrent cervical–occipital fixation was carried out (Figure 4g).

### 5.6. Case 6: (C4Di2Vi)

**Figure 5:**
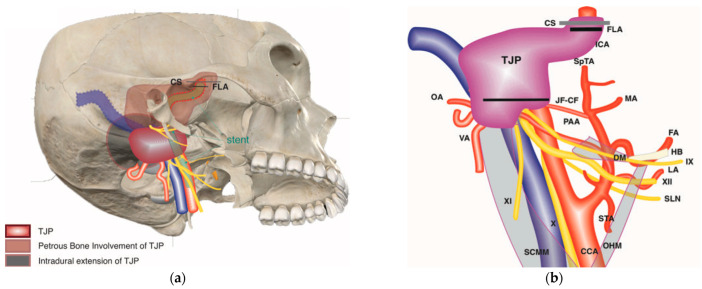

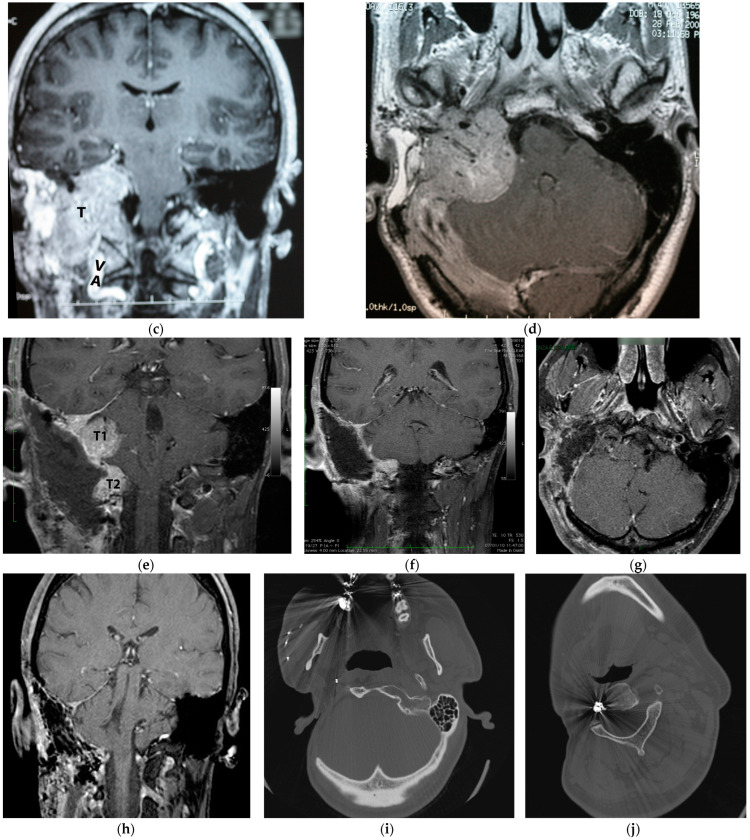
(Case 6) (**a**,**b**) Right-sided tympanojugular PGL (C4Di2Vi). (**c**) Coronal Gd-enhanced T1-weighted magnetic resonance imaging (MRI) shows a large residual tumor extending to the intradural space involving the vertebral artery. (**d**) Axial Gd-enhanced T1-weighted MRI shows a large residual tumor extending to the intradural space, involving transverse sinus ipsilaterally up to the torcula. (**e**) Coronal Gd-enhanced T1-weighted MRI after first-stage tumor removal. Note the residual tumor: T1 is in the cerebellopontine angle, and T2 is in the foramen magnum. (**f**) Coronal Gd-enhanced T1-weighted MRI after second-stage tumor removal, which shows a small residual tumor at the foramen magnum. (**g**,**h**) Axial and coronal Gd-enhanced T1-weighted MRI after third-stage tumor removal, which revealed total tumor removal at the level of the foramen magnum. (**i**,**j**) Axial CT scan after the third stage shows a coil at the internal carotid and vertebral arteries that are used for artery occlusion.

A 41-year-old man with a right-sided TJP (C4Di2Vi) who had undergone three prior surgeries with partial tumor excision came to our facility. He had HB-IV facial palsy, cranial nerve palsies IX and Xll, and a dead ear on the same side at the time of presentation. The management was to stage the tumor resection (Figure 5). During the first stage in 2008, he underwent extradural tumor removal through a modified ITFA-A with transcondylar extension, and the FN and LCNs; IX, X, XI, and XII were sacrificed. During the second stage in 2009, through a modified transcochlear approach (MTCA), he underwent the removal of remnant extradural and intradural tumors. Following the second-stage surgery, coils were used to permanently occlude the tumor-involved vertebral artery (Figure 5j). The third stage was conducted in 2010 through an extreme lateral approach to remove the tumor between the foramen magnum and vertebral artery and drill around the foramen magnum until healthy bone was reached. During the fourth stage in 2011, he underwent a static facial reanimation through a mid-facelift procedure. After receiving gamma knife treatment in 2014 for a sub-centimetric remnant in the cavernous sinus, the tumor remained stable. The most recent follow-up was in 2023.

## 6. Results

Out of the 262 individuals with a pathologically proven TJP treated surgically by the senior author (M.S.), six patients made up the study group. There were four (66.6%) males and two (33.3%) females. At the time of operation, the mean age was 40.5 years old (range: 25–53). There were four (66.6%) tumors on the right side and two (33.3%) on the left. Table 4 includes the pertinent patient demographics, tumor locations, specific surgical techniques performed in each case, and surgical treatment outcomes for the six patients included in the case series. The most common symptoms at the time of presentation were glossal atrophy (GA) (83.3%), hearing loss (66.6%), pulsating tinnitus (66.6%), dysphonia (66.6%), dysphagia (66.6%), shoulder weakness (33.3%), and diplopia (16.6%) (Table 2).

One patient had paralysis of five cranial nerves (VII, IX, X, XI, and XII); one patient had paralysis of nerves IX, X, XI, and XII; and two other patients presented multiple paralysis of nerves (X, XI, XII, X, and XII). The last patient had palsy of the VI cranial nerve. The FN nerve function; preoperative, immediate postoperative, and during the last follow-up is summarized in (Table 3).

All cases showed intradural involvement by the tumor mass (D). Two cases had multiple concomitant paragangliomas (TJ + vagal). All patients were treated with embolization 48 h before surgery. In three cases, it was necessary to place a stent from the carotid bifurcation to the pre-cavernous internal carotid artery at the level of the ICA to proceed with the surgery, and in two cases, the vertebral artery was closed by a coil (Table 4).

**Table 4:** Patient demographics, PGL classification, type of surgery, and postoperative complications.

Patient Demographics, PGL Classification, Type of Surgery, and Postoperative Complications											
Patient	Age at Surgery (y)	Sex	Side	Pre-op. CN Deficits	Class of Tumor	Other Surgeries/ Treatments	Embolization or Stenting	Surgical Approach	New CN Deficits	Tumor Resection	Follow-Up (m)
1	25	M	L	IX, X, XI, and XII	C4Di2	3	Stent of left ICA + PBO	− ITFA-A extended to the parotid gland (extradural portion); − POTS + TLA + sural graft (intradural portion).	-	Total	224
2	40	M	R	IX, X, XI, and XII	C3Di1 + vagal	1 surgery for CB PGL (left side)	Left ICA closed in another surgery and embolization involving stent of right ICA	− ITFA-A	-	Total	95
3	32	F	R	VI	C4Di2Vi	-	Absence of contralateral compensation and embolization involving stent of right-ICA	− ITFA-A (1996) (extradural portion); − POTS (1998) (partial intradural portion); − POTS (2004) recurrence with transcondylar extension (extradural portion); − POTS (2004) (intradural portion);	IX, X, and XII	Total	336
4	53	F	L	X and XII	C3Di2 + vagal	-	Embolization	− ITFA-A (extradural portion); − POTS (intradural portion).	XI	Total	38
5	53	M	R	X, XI, and XII	C3Di2Vi	Radiation therapy	Embolization	− ITFA-A transcondylar; − ITFA + TC (intradural portion); − Transcochlear (clivus portion).	IX	Total	35
6	41	M	R	XII	C4Di2Vi	1 surgery in 2002	Embolization	− ITFA-A + TC (extradural portion); − MTCA (intra- and extradural portion); − EL (foramen magnum); − Facial reanimation.	IX, X, and XI	Residual gamma knife (stable)	145

Abbreviation: CB, carotid body tumor; PBO, preoperative balloon occlusion; ITFA-A, Infratemporal Fossa Approach, Type A; TC, transcondylar extension; EL, extreme lateral approach; POTS, petro-occipital trans-sigmoid; TLA, trans-labyrinthine approach; MTCA, modified transcochlear approach.

## 7. Discussion

Six difficult TJPs make up the current series. Since our center is recognized as one of the world’s top referral sites for lateral skull-base conditions, including TJPs, all six cases were referred with advanced tumor stages. Some of them underwent multiple operations at different locations throughout this period.

The mainstay of managing TJPs is total surgical extirpation. However, most of the time, it is not easy to achieve this surgical goal due to the tumor’s location in relation to vital neurovascular structures and local infiltrative behaviors [19]. Other options in the management of TJPs include partial excision with adjuvant RTs, stereotactic radiosurgery [20], and active surveillance (wait and scan) once total surgical removal is not possible due to the patient’s condition or due to the involvement of critical structures such as the cavernous sinus, AICA, brain parenchyma, and basilar artery [21].

The last few decades have seen a major increase in the use of preoperative direct and intra-arterial embolizations, particularly with polyvinyl alcohol particles, in the treatment of paragangliomas, which is mostly due to developments in high-resolution imaging and super-selective catheterization techniques. The effectiveness of blocking particular tumoral feeders of PGL tumors has significantly increased with the development of super-selective embolization techniques [22,23]. Compared to the surgical resection strategy without embolization, embolizing PGL preoperatively provides a number of advantages. Preoperative embolization has been shown, in numerous studies, to reduce the incidence of bleeding events and blood transfusions after surgery [24,25,26]. A frequent observation after embolization is a reduction in the tumor size, which raises the possibility of a technically successful excision and could shorten the duration of the procedure [27]. Tumors that were once regarded as inoperable in the old days can now be managed safely. Nevertheless, some TJP cases are too demanding to treat [2,7,8,9,10,11,12]. Examples of such cases include large tumors; those with a massive intradural extension; tumors involving the cavernous sinus and the ICA or VA and single carotid artery; tumors with the dominant or unilateral sigmoid sinus on the tumor side; previously operated or irradiated tumors; as well as bilateral or multiple tumors [1].

Broadly, the subtype C3 and C4 TJP tumors in Fisch and colleagues’ classification are considered large tumors. When TJPs enlarge, they either spread to the petrous apex through an invasion of the carotid canal, or they spread through the medial wall of the jugular bulb into the intradural space, which involves the LCNs [2,28]. As a rule, the classic ITFA can be used for class C1 and certain class C2 tumors, while the class C2–C4 subtype of TJPs—can be managed with the ITFA-A with a transcondylar trans-tubercular extension [1]. However, an additional extension such as the modified transcochlear or extreme lateral transcondylar approach might be required when there is an invasion of the clivus or foramen magnum by the tumor. Furthermore, there is a relationship between the size of the tumor and the probability of maintaining the LCN function. A larger tumor reduces the likelihood of maintaining the LCN function [29,30].

When the intradural extension is large, the frequency of the LCN’s involvement and the brainstem compression will be high. It is quite difficult to assess the extent of dural involvement through preoperative radiology or intraoperative findings. If a small area of the dura is invaded, it can be easily removed and managed immediately, whereas, if there is massive dural involvement, it can be managed through staged tumor removal. Widespread intradural recurrence might result from any undetected involvement. Many authors have proposed that single-staged tumor removals are suitable even if there is extensive dural and intradural involvement [3,5,31], but we observed that the risk of cerebrospinal fluid (CSF) leaks is high when resecting a large intradural (Di2) tumor as compared with staged tumor resection, and this is postulated due to several factors: First, the risk of sacrificing LCNs is high when dealing with a large intradural tumor (Di2), which subsequently leads to an increased risk of CSF leaks secondary to increased intracranial pressure from severe aspiration and interactable coughing [28]. Second, larger skull base and dural defects, as well as the direct communication between the intradural and neck space, occur when dealing with Di2 tumors, which in turn leads to a higher incidence of CSF leaks. Thus, for Di2 TJPs, we favor staged tumor removals to prevent postoperative CSF leakage and single-stage removals for tumors with a smaller intradural extension, which is consistent with the practice in other studies [2,15]. Additionally, 5.2% (3.8–33.3) is the average rate of postoperative CSF leaking in a single-stage surgery [5,10,32,33]. In addition, over the years, we have observed that intradural tumor removal is relatively easy during the second stage because the residual tumor has been partly devascularized during the initial stage. A dissection and conservation of the LCN function during the second stage will be more easily achieved, and a preoperative embolization of the feeder vessel during subsequent stages will further help to lower the amount of operative blood loss.

In the second stage, the patient’s hearing level and the size and location of the tumor are the main determinant factors for the approach selection. Although the POTS approach [15,34] is the favorable approach among a vast majority of patients, an MTCA or an extreme lateral transcondylar approach may be utilized as well.

The presence of a normal preoperative LCN function does not exclude an intraoperative neural invasion by the tumor [30]. In fact, a tumor extension beyond the dura generally suggests the involvement of the LCN [21]. It has been suggested by some authors to leave the piece of tumor that is attached to LCNs to preserve the function of the LCN, especially in elderly patients [35]. However, in young patients, if total removal is technically reasonable, it is preferred to sacrifice tumor-infiltrated LCNs to avoid recurrence. Most of our patients for whom the LCNs have been sacrificed are well compensated even without postoperative rehabilitation.

If the TJP involves the cavernous sinus, it is recommended to intentionally leave that part of the tumor to avoid sacrificing the cranial nerves III, IV, and VI to maintain ocular mobility. Postoperatively, stereotactic radiotherapy can be given as an adjuvant therapy to control the rate of tumor growth. We have two cases involving the cavernous sinus, and both of them are stable, with no growth of the residual tumor on the cavernous sinus in the most recent follow-up MRI [6].

The modified transcochlear type D or extreme lateral approach is used when there is an involvement of the foramen magnum and lower clivus [10,31]. To avoid leaving the residual tumor, the clivus should be drilled until healthy bone appears because of the infiltrative character of TJPs.

Due to its close anatomical proximity, the ICA is frequently affected by TJPs. The surgical planning and whether the tumor is operable is determined by the degree of the arterial wall involvement by the tumor. When appropriate, the tumor must be dissected from the arterial wall, which can be achieved by either a subperiosteal or subadventitial dissection. A subperiosteal dissection is achieved by finding and dissecting the cleavage plane between the periosteum of the carotid artery canal and the adventitia of the ICA [2]. This method of dissection is appropriate when a tumor involves only the periosteum and is relatively safe and easier in the vertical part of the petrous carotid artery than in the horizontal part of it, as the former has a thicker wall and is more easily accessed. A subadventitial dissection, on the other hand, is a dissection between the adventitia and the muscular layer. This is a delicate step during the surgery and should be carried out with caution, as it may lead to tears in the wall of the ICA. The thickness of the vertical segment of the petrous ICA is around 1.5–2.0 mm, with the adventitia being ~1 mm thick; meanwhile, there is no adventitia in the horizontal segment [17]. Therefore, a subadventitial dissection cannot be carried out at the level of the horizontal part of the petrous ICA.

When the tumor fully encases the artery with extreme narrowing, as evidenced by arteriography, any intervention without sufficient preoperative endovascular control of the ICA may lead to a massive hemorrhage, residual tumor, or stroke [13,35,36,37,38]. Whenever there is adequate collateral blood supply, a preoperative balloon occlusion is carried out if the tumor infilters the wall of the artery. But if the collateral blood supply is inadequate, we usually reinforce the artery with intraluminal stenting [38], which in turn increases the thickness of and strengthens the arterial wall that allows for generating a dissection plane at the outer surface of the stent to excise the tumor completely without risking an inadvertent carotid rupture. With the advent of these new techniques (endovascular stenting and preoperative balloon occlusion), selected patients who were regarded for a subtotal resection can be safely re-evaluated for a total removal of the tumor.

In order to remove the tumor totally, it is critical to address the ICA preoperatively. Options for those patients with a single carotid artery at the side of the lesion include the following: either wait and scan (especially among elderly patients); an incomplete tumor removal with adjuvant radiotherapy; and total tumor removal (following intraluminal stenting or by utilizing bypass surgery), but the later poses significant risks of cerebral ischemia and strokes. Preoperative intraluminal stenting, however, may be the optimal choice.

So far, at Gruppo Otologico, we have managed two cases with a single ICA on the lesion side with no postoperative complications. One of them is a 55-year-old woman with a previous history of a contralateral ICA occlusion, which had been managed for an intracranial aneurysm, that was found to have a suspected tumor infiltration of the vertical segment of the ICA, as evidenced by angiography. During the observation period, the tumor was noted to have grown, prompting the discussion of a preoperative high-flow carotid artery bypass as a potential management strategy. Ultimately, the surgical team’s familiarity and positive experiences with stent insertions in similar cases led them to favor this approach over the bypass procedure, and they proceeded with the stent placement before the tumor resection. Given the patient’s advanced age, the substantial extent of the tumor, and the presence of bothersome brainstem compression, the treatment team determined that radiotherapy would not be an appropriate or effective treatment option in this case. The successful placement of the stent enabled the surgical team to subsequently accomplish a complete gross total removal of the tumor. The patients experienced no postoperative complications and were free from disease at 15–20-year follow-ups.

In the case of extradural and/or intradural vertebral artery involvement (Ve/i), we added a new subclass to the Fisch classification of TJPs and removed the De component. This was performed because we observed that, in the case of class D tumors, the tumor had already penetrated the cranial cavity in both the De and Di components and that the general surgical approach for excisions used for both components was nearly the same [16,39].

We discovered that 8 out of the 230 individuals with TJPs had vertebral artery involvement. Six individuals had intradural VA involvement, and one patient had an involvement of the extradural artery. In one patient, both the intradural and extradural VAs were affected at the same time. Out of the eight, seven had surgeries. Through a microdissection, we were able to remove the tumor from the artery in five of the seven patients. A preoperative occlusion was conducted on two patients: one with coils and the other with a balloon. One patient out of the seven underwent surgery to remove a subtotal tumor from the vertebral artery. Tumors surrounding the VA were removed without any significant issues. A gross complete tumor removal without major surgical complications was made possible by the VA’s preoperative examination and intervention.

Because a TJP typically results from jugular bulb adventitia, surgical procedures to remove the tumor typically involve closing the sigmoid sinus and ligating the jugular vein. On the other hand, venous congestion, intracranial hypertension, and brain edema may result from a closure of the dominant or sole sigmoid sinus on the lesion side [10]. Therefore, it is crucial to evaluate the brain’s venous drainage system prior to the surgery, especially the ipsilateral mastoid emissary vein and condylar vein. The sigmoid sinus should be closed distal to the mastoid emissary vein’s exit if their diameters are more than normal. Otherwise, they should be left undisturbed. In cases where the patient lacks adequate collateral venous drainage or where it is not possible to preserve collateral venous drainage, more conservative measures, such as stereotactic radiosurgery, subtotal resection with a preserved sigmoid sinus, or “wait and scan”, are advised.

The patient’s life is directly and significantly impacted by the management of bilateral paragangliomas. One should never undervalue the significance of neuronal preservation, because bilateral deficiencies in LCNs may occur. The surgeon’s primary focus during the initial stage of the surgery should be on removing the tumor while maintaining the LCN’s functionality [5,40]. If a larger tumor has an LCN deficit on its side, it should be removed before managing the smaller tumor with radiation therapy or a follow-up. Similarly, a smaller tumor should be removed first if a patient has an LCN deficit on that side, and then the larger tumor on the contralateral side can be monitored. If the tumor grows, it will be managed through a partial removal of the tumor, while maintaining the LCN with adjuvant radiotherapy [1].

However, if both paragangliomas, in the case of bilateral paragangliomas with a normal LCN function, are of the same size, they can be monitored by serial MRIs in addition to the surgery or radiation therapy recommended for tumors that show growth. Surgery is carried out on the side of the larger tumor if the two tumors differ in size to maintain the LCN function. If the function of the LCN is preserved postoperatively, surgery can be considered for the contralateral side as well. On the other hand, if the LCNs are sacrificed, the contralateral tumor needs to be monitored using MRIs regularly and treated with radiation if growth is seen. When dissecting the tumor, it is crucial to protect the jugular bulb’s medial wall to maintain the LCN function [5,41].

The cervical section of the vagus nerve should always be recognized and preserved during ITFA-A. Conversely, it is advised to remove both lesions at the same time when vagal paragangliomas and TJPs are present on the same side.

When treating infiltrative skull-base lesions, it is important to remember that persistent or recurring tumors are always a possibility. To ascertain whether radiation, revision surgery, or wait and scan is the best course of action, a thorough radiological appraisal is necessary. It is more difficult to perform revision surgeries because there are no typical tissue planes or surgical landmarks. A history of prior radiation therapy or surgery increases the risk of CSF leaks, injury to LCNs, and damage to the facial nerve [3,10,11,13]. Because irradiated tissue is often hard, removing and dissecting tumors can be challenging [13]. The carotid canal is the most frequent location of recurrence in TJPs, and injuries to the ICA are more likely following prior dissections. Consequently, as was already noted, preoperative treatments of the ICA are quite important.

We performed an anterior FN rerouting with the appropriate extension in all cases during an ITFA. Conservative care of the external auditory canal and FN is not appropriate during revision surgeries. Thirteen individuals in one of our series had received prior therapy; eight of these had undergone different mastoidectomies without rerouting the FN, and following the surgery, a remnant tumor was found under the nerve and surrounding the carotid artery. This was probably caused by either partial excision surrounding the ICA or a lack of facial nerve rerouting.

As a result of the anterior facial nerve rerouting as part of the ITFA-A, the patients experienced a new-onset or worsening facial weakness, which improved within a year (Table 2). One patient experienced a postoperative CSF leak, which was managed with a muscle graft and facia lata to seal the leak. Four patients experienced new-onset LCN palsies (Table 4), which the patients tolerated. None of the six patients had embolization-related complications.

In some instances, complete tumor removal requires a balloon occlusion or intraluminal stenting of the ICA. Due to the infiltrative nature of paragangliomas, wide bone drilling is necessary to prevent residual tumors; preservation of LCNs infiltrated by the tumor should not be attempted, particularly among younger patients.

## 8. Conclusions

Having one of the biggest series in TJP management has allowed us to draw several conclusions. The cornerstone of this type of surgery is IFTA-A. By rerouting the facial nerve anteriorly, we can lower the likelihood of recurrence and enhance the management of the carotid artery. Planning requires a thorough investigation of the brain’s hemodynamics. To prevent jeopardizing the patient’s life in situations where there are bilateral tumors, every effort should be taken to maintain the LCN function, if only unilaterally. Complete removal is facilitated by appropriate preoperative endovascular interventions such as PBOT or stenting. Staged removal is advised in cases with a significant intradural component in order to lower the danger of CSF leakage and to make the second stage’s intradural tumor removal more manageable and technically straightforward. A preoperative evaluation and intervention, along with the careful management of aggravating variables, can significantly reduce surgical morbidity with a high chance of gross complete removal. Some tumor expansions, such as those enclosing the posterior circulation vasculature, those extending into the cavernous sinus, and those exhibiting a parenchymal brain invasion, are nonetheless incurable without unacceptably high morbidities.

## Figures and Tables

**Table 1:** Modified Fisch classification of TJ PGLs.

**Class C**	Tumors extending beyond the tympano-mastoid cavity, destroying the bone of the infra-labyrinthine and apical compartment of the temporal bone and involving the carotid canal	
	C1	Tumors with limited involvement of the vertical portion of the carotid canal
	C2	Tumors invading the vertical portion of the carotid canal
	C3	Tumors with invasions of the horizontal portion of the carotid canal
	C4	Tumors reaching the anterior foramen lacerum
**Class D**	Tumors with intracranial extension	
	Di1	Tumors with up to 2 cm of intradural extension
	Di2	Tumors with more than 2 cm of intradural extension
	Di3	Tumors with an inoperable intradural extension
**Class V**	Tumors involving the VA	
	Ve	Tumors involving the extradural VA
	Vi	Tumors involving the intradural VA

**Table 2:** Signs and symptoms at presentation at our center.

Symptoms and Signs	No. of Patients (%)
Hearing loss	4 (66.6)
Pulsating tinnitus	4 (66.6)
Vertigo	2 (33.3)
Dysphonia	4 (66.6)
Dysphagia	4 (66.6)
Glossal atrophy	5 (83.3)
Shoulder weakness	3 (50)
Diplopia	1 (16.6)

**Table 3:** Facial nerve function.

Preoperative, Immediate Postoperative, and Final Facial Nerve Function According to House–Brackmann Scale			
Patient	Preoperative (HB) *	Immediately Postoperative (HB)	At Last Follow-Up (HB)
1	III	VI	IV
2	I	VI	III
3	I	VI	I
4	I	VI	III
5	I	VI	III
6	IV	VI	III (after V–VII anastomosis)

* Status of VII cranial nerve (HB) before surgery at Gruppo Otologico.

## Data Availability

The data presented in this study are available on request from the corresponding author. The data are not publicly available due to the fact that the patient data include preoperative and postoperative follow-up imaging and investigations, which were all archived at Gruppo Otologico.

## Supplementary Materials

The following supporting information can be downloaded at: https://www.mdpi.com/article/10.3390/brainsci14080745/s1.
